# Lymphoepithelial Cyst of Parotid in an Immunocompetent Patient with Chronic Otitis Media

**DOI:** 10.1155/2017/5169364

**Published:** 2017-07-30

**Authors:** Meera Niranjan Khadilkar, Vishnu Prasad, Vijendra Shenoy Santhoor, M. P. Kamath, Haneesh Domah

**Affiliations:** Department of ENT, Kasturba Medical College, Manipal University, Mangalore 575001, India

## Abstract

Lymphoepithelial cysts of parotid are known to occur in HIV patients. In the present report, lymphoepithelial cyst of parotid was diagnosed in a middle aged immunocompetent patient, along with chronic otitis media. The source of infection and treatment options are summarized.

## 1. Introduction 

Benign lymphoepithelial cysts are a widely accepted cause of parotid gland swelling in human immunodeficiency virus (HIV) patients and are pathognomonic for HIV. They often grow to be extremely large, resulting in physical deformity and gross facial asymmetry [1]. In the present study, lymphoepithelial cyst of parotid was diagnosed in a middle aged immunocompetent patient, along with chronic otitis media.

## 2. Case Report 

**Figure 1:**
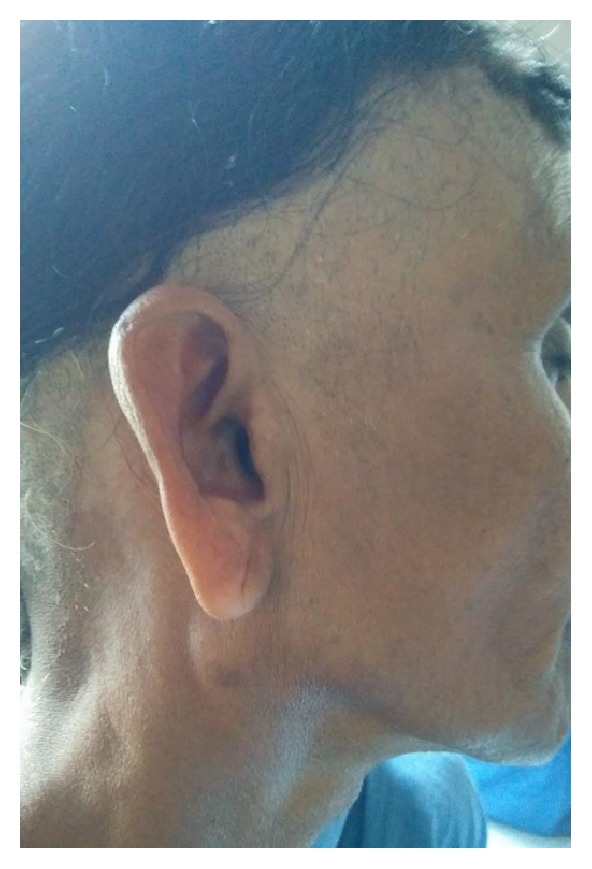
Figure showing right parotid swelling.

**Figure 2:**
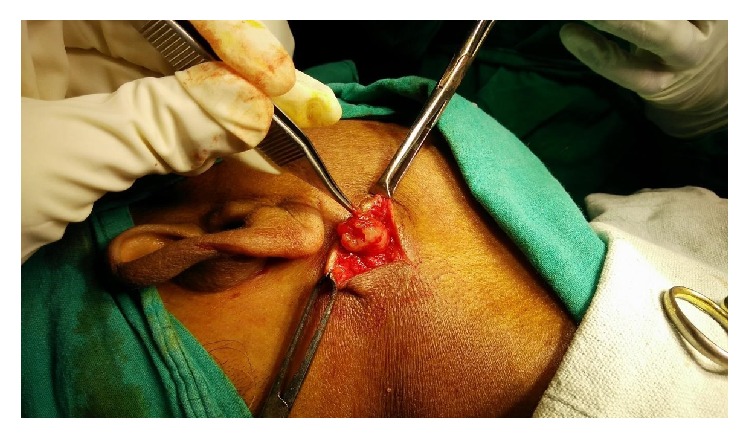
Intraoperative photograph showing enucleation of the parotid cyst.

A 55-year-old lady presented to the ENT OPD with a swelling below her right ear since four months. It was insidious in onset and gradually progressed in size. Incidentally, she also gave history of occasional right sided ear discharge since four months. The swelling was not associated with pain or change in size during meals. Examination showed a 2 × 2 cm mobile swelling in front of and below the right ear, which was smooth, soft, and nontender, with no local rise of temperature (Figure 1). Stenson's duct opening was normal. Left parotid was normal. Right external auditory canal was normal. Right tympanic membrane had a small central perforation. Right level two cervical lymph nodes were palpable. Ultrasound scanning of the cheek and fine needle aspiration cytology revealed right parotid cyst. She surprisingly tested negative for HIV. Enucleation of the cyst with a cuff of normal parotid tissue was planned under general anaesthesia, and the cyst was delivered in toto (Figure 2). Histopathological report confirmed the presence of lymphoepithelial cyst. Postoperative period was uneventful, with normal facial nerve function. She was advised to undergo tympanoplasty at a later date.

## 3. Discussion 

BLECs are solitary or multiple cysts within lymph nodes trapped in the parotid gland during embryogenesis, representing cystic degeneration of salivary gland inclusions within the intraparotid lymph nodes, mainly located along the tail of the parotid, thereby predisposing this part of the gland [2, 3]. They are slow growing in nature, typically seen in HIV positive adults. It is unusual to find them in non-HIV individuals [4].

The pathogenesis of this disease is debated. HIV-infected cells migrate into the parotid, resulting in lymphoid proliferation and metaplasia in the salivary ducts. Ductal obstruction secondary to cellular proliferation leads to dilatation and cyst formation [2, 5]. Another concept is that reactive lymphoproliferation occurs in the lymph nodes of the parotid. The parotid glandular epithelium gets trapped in normal intraparotid lymph nodes causing cystic enlargement [2, 3]. Even though the mechanism of BLEC formation in immunocompetent individuals is not clear, it can be postulated that a similar process may be responsible as a result of lymphoid proliferation in viral infections other than HIV. In the present study, the presence of chronic otitis media indicates a long standing infection and reactive hyperplasia in the draining lymph nodes in the vicinity; the cyst could be the consequence of infection of the intraparotid lymph nodes.

With the emergence of HIV epidemic, the incidence of parotid BLEC has increased to an estimated 3% to 6% in HIV positive adults [6]. However, the exact incidence of the same in immunocompetent individuals is not known.

Diagnosis of BLEC is based on history, physical examination, and fine needle aspiration biopsy [7, 8]. Treatment of this particular pathology has been widely discussed in the literature. The conservative approach includes cyst decompression by aspirating the fluid out thereby reducing the pressure. This should be considered in an immunodeficient patient, in which the surgical management is clearly outweighed by the risk. Other methods include external radiotherapy, sclerotherapy, and HAART, in immunocompromised patients. Definitive treatment is complete enucleation of the cyst along with the excision of the involved gland.

Most patients are completely cured by excision and never get a recurrence [1, 3].

## 4. Conclusion

Lymphoepithelial lesions can manifest as cystic parotid swellings even in immunocompetent individuals, in whom the possible source of infection could be attributed to chronic otitis media, spreading to the intraparotid lymph nodes. Surgical option of enucleation of cyst with a cuff of normal parotid tissue forms the mainstay of treatment in such individuals.
